# Therapeutic Pearls at Random Strung

**Published:** 1881-04

**Authors:** 


### THERAPEUTIC PEARLS AT RANDOM STRUNG.

### Gonorrhœa

1st.

*Oil of Erigeron*, an oil obtained from the Erigeron Canadense, has been found to act with unvarying success in ten drop doses, on a little sugar, and three hours later a full dose of Spts. Nitrous Aether in infusion of Althea; and so on every three hours until the urethral inflammation is subdued. The treatment is recommended to be preceeded by an active Saline Cathartic.

2ND.

| | | |
|---|---|---|
| ℞ Oil Cubebs. | | |
| " Copaiba, | . . . . . . | aa ʒ iv |
| Chloroform, | . . . . . . . | ʒ ij |
| Creosote, | . . . . . . . . | gtt v |
| Pulv. G. Acae, | . . . . . | ʒ ss |
| Water, | . . . . . . . | ℥ iv M. |

Dose: Teaspoonful every four hours.

3RD.

OIL OF YELLOW SANDAL WOOD (*Ol. sant. flav*).

The *above Oil*, given in doses of 15 to 30 drops in *peppermint* water, three or four times a day, is said to act almost specifically in Gonorrhœa, Blenorrhœa, &c.

4TH.

| | | |
|---|---|---|
| ℞ Sulphate Zinc, | . . . . . . | ʒ i |
| Pulv. Alum, | . . . . . . . | ʒ i |
| Sulph. Morphia, | . . . . . | ℈ ss |
| Water, | . . . . . . . . | Oj M. |

Inject into the urethra three or four times per day and retain it in the urethra a minute or two each time. Pass the urine immediately before injecting. The injection may be repeated *once or twice* at those periods with advantage.

5TH.

| | | |
|---|---|---|
| ℞ Sulph. Zinc, | | |
| Aceta Morphia, | . . . . . | aa ℈ i |
| Agnæ Rosar, | . . . . . . | f ℥ viij M. |

Used as foregoing, obeying the same rules.

6TH.

HYDRASTIN IN GONORRHŒA.

Keep the bowels open with a saline aperient. In the first stage for 4 or 5 days, and use the following injection:

| | | |
|---|---|---|
| ℞ Hydrastin, | . . . . . . . | ʒ i |
| Acacia Mucilage, | . . . . . | ℥ iv |
| Solution Morphia, (Magendies) | . . | ʒ ij |
| | | M. |

Use as an injection 3 times a day. This injection may be used in every stage of the disease, even when the inflammation runs high. The urine should always be voided immediately before the injection is used.

### Gouty Diathesis.

We give below an elegant prescription suitable for constant use by persons suffering from troubles arising from the gouty diathesis. By Prof. J. S. Welford, M. D.:

℞ Carbonate sodæ, . . . . gr. iiiss.
Carbonate Lithia, . . . . . gr. iss.
Carbonate acid water, . . . . ℥ j.
M. S. Dose.

### Heart Tonic and Diuretic.

In the general Anasarca and Ascites that sometimes accompany disease of the heart and functional derangement of the liver, this formula is perhaps without a rival, viz:

℞ Potassii Acetas,
Tinct. Card. Com, . . . . . aa ℥ j.
" Digitalis. . . . . . . ℥ ss.
Aqua Det. q. s., . . . . . ℥ viij.
S. M.

℥ ss four or five times daily, prore nata. The bowels are to be kept free, in case the solution fails to do so, by the use of ⅛ gr. pills of eleterium once or twice daily.

### Hair Dye.

A new hair dye without noxious properties.

Subnitrate Bismuth, 10 parts, and glycerine 150 parts, are mixed in a glass vessel and heated in a water bath; solution of potassa is then added in small quantities, and with continued agitation, until a clear solution has been obtained, to which a concentrated solution of citric acid is added, until merely a slight alkaline reaction is observed.

Enough orange flower water is added to make the whole weigh 300 parts; the addition of a small quantity of solution of an orange-color completes the preparation.

### Another Hair Dye.

The Greeks and Persians use a hair dye made from *walnut rinds.* Kurts recommends that it should be prepared by boiling the green rinds in water, and adding *alum* to the decoction.

### Kerosene Horse Liniment.

A correspondent sends the following: "It is not as generally known as it ought to be, that kerosene oil is one of the very best remedies for strains, sprains or bruises that can be applied to the flesh of beasts. I knew an instance in which a young colt got cast on its back in a manger; when taken out it was utterly unable to stand or move its hind limbs, and so continued for some time, when two or three applications of the oil, twelve hours between, completely restored it. This and similar facts led me, when studying drugs several years ago, to concoct the following, which I named kerosene liniment: "Kerosene oil, one ounce; aromat spirits ammonia, three drams; tinc. stramonii, two drams; tinc. opii, two drachms; tinc. arnica, two drams; ol. origanum, two drams; ol, menthe, twenty drops; chloroform, one dram; spirits camphor, two drams. This article is incomparably superior to anything of its kind for bruises, soreness of the muscles from any cause, or nervous pains. Its value, however, will best be known by its use. Many a prescription has been sold for $50, which, compared with this, was not worth the paper it was written on. Equally good for beasts or men.

### Hemorrhage.

Tincture of *Erigeron Canadense.* Dose, one drop in a little water every minute or two in cases of venous or capillary hemorrhages.

### Hoarseness and Temporary Loss of Voice.

Dissolve slowly in the mouth, and swallow as dissolved, a piece of *Borax,* 3 or 4 grs., three or four times a day. *Nitrate of Potash* taken in like manner on retiring at night, will facilitate the cure where borax alone is insufficient. Care must be exercised the day *after* the use of the *salt petre,* to avoid taking cold.

### Falling of the Hair.

The most satisfactory treatment of Alopecia Pityrodes is the use of Tannin ℈jv, Adipis ℥j. Under the use of this preparation the hair does not fall so much, and the increase is more obvious. Oleum Sabinæ gtt, 5 to 30, to Lard ℥ i has this effect still more decidedly, but causes the hair to become harsh and stiff, and of a dirty color, and has an unpleasant odor; besides this, it sometimes causes severe headache.

### HEMORRHOIDS.

No. 1.

℞ Sulph. Morphia, . . . . . grs. iij

Extract Stramonium, . . . . . ʒ ss

Olive Oil, . . . . . . . . ʒ i

Carbonate Lead, . . . . . . ʒ i

Lard Cerate, . . . . . . . ʒ iij

Rub the extract, if not uniformly soft, with a few drops of water; add the powders and olive oil, and rub until perfectly smooth, then incorporate them with the cerate.

No. 2.

℞ Glycerine, . . . . . . . ℥ i
Carbolic Acid. . . . . . grs. iv to x.

(Tannin, grs. v to x may be added or not as desired).

Mix. Apply on a piece of cotton to the part, after bathing it in cold water. A few grains of Morphia may be added when necessary to allay pain.

No. 3.

Lime Water thrown into the rectum to the amount of three or four tablespoonfuls, and allowed to remain after each evacuation; is said, by Prof. Agnew, to be *the* remedy. Superior to all others in affording relief, and often curing internal piles.

No. 4.

The internal use of the powdered leaf of the Wild Germandra, (Tucrium Scordium,) in doses of from 15 to 20 grains, three times a day in water, is said to exert highly beneficial action in the relief of the pruritus, and all other unpleasantness in hemorrhoids, in a short time.

